# Lignin Nanoparticles: Transforming Environmental Remediation

**DOI:** 10.3390/nano14181541

**Published:** 2024-09-23

**Authors:** Pirzada Khan, Sajid Ali, Rahmatullah Jan, Kyung-Min Kim

**Affiliations:** 1 Biotechnology Research Institute, Chinese Academy of Agricultural Sciences, Beijing 100081, China; pirzada_111@yahoo.com; 2 Department of Horticulture and Life Science, Yeungnam University, Gyeongsan 38541, Republic of Korea; drsajid@yu.ac.kr; 3 Coastal Agriculture Research Institute, Kyungpook National University, Daegu 41566, Republic of Korea; 4 Department of Applied Biosciences, Graduate School, Kyungpook National University, Daegu 41566, Republic of Korea

**Keywords:** lignin nanoparticles, environmental remediation, sustainable paradigms, water purification, eco toxicological assessments

## Abstract

In the face of escalating environmental challenges driven by human activities, the quest for innovative solutions to counter pollution, contamination, and ecological degradation has gained paramount importance. Traditional approaches to environmental remediation often fall short in addressing the complexity and scale of modern-day environmental problems. As industries transition towards sustainable paradigms, the exploration of novel materials and technologies becomes crucial. Lignin nanoparticles have emerged as a promising avenue of exploration in this context. Once considered a mere byproduct, lignin’s unique properties and versatile functional groups have propelled it to the forefront of environmental remediation research. This review paper delves into the resurgence of lignin from an environmental perspective, examining its pivotal role in carbon cycling and its potential to address various environmental challenges. The paper extensively discusses the synthesis, properties, and applications of lignin nanoparticles in diverse fields such as water purification and soil remediation. Moreover, it highlights the challenges associated with nanoparticle deployment, ranging from Eco toxicological assessments to scalability issues. Multidisciplinary collaboration and integration of research findings with real-world applications are emphasized as critical factors for unlocking the transformative potential of lignin nanoparticles. Ultimately, this review underscores lignin nanoparticles as beacons of hope in the pursuit of cleaner, healthier, and more harmonious coexistence between humanity and nature through innovative environmental remediation strategies.

## 1. Introduction

In an era where environmental challenges loom large, scientific research continually seeks innovative solutions to address the escalating issues of pollution, contamination, and ecological degradation. The consequences of human activities, from rapid urbanization to industrialization, have left an indelible mark on the Earth’s ecosystems [1]. As we grapple with the ramifications of anthropogenic actions, the imperative to develop sustainable and effective strategies for environmental restoration has never been more pressing [2].

The depletion of natural resources, the degradation of air and water quality, and the loss of biodiversity are but a few of the profound consequences of humanity’s relentless pursuit of progress [3]. The exponential growth of industrialization, driven by technological advancements, has significantly altered the balance of ecosystems. As a result, the Earth now bears the scars of indiscriminate exploitation and pollution, leading to global challenges that transcend geographical and disciplinary boundaries [4]. Amid these challenges, there is a growing realization that traditional approaches to environmental remediation are often insufficient to address the complexity and scale of modern environmental problems [5]. The linear models of resource extraction, utilization, and disposal have demonstrated their limitations, ushering in an era of transition toward circular and sustainable paradigms [6]. Central to this transition is the exploration of novel materials and technologies that possess the potential to rectify the damage incurred by human activities [7]. In this quest for innovative solutions, one particular avenue of exploration has garnered increasing attention within the scientific community: the utilization of lignin nanoparticles for environmental remediation [8]. Lignin, often regarded as a mere byproduct of the paper and biofuel industries, has undergone a remarkable transformation in recent years. Its intricate structure, remarkable abundance, and diverse functional groups have captured the curiosity of researchers seeking novel materials capable of effecting positive change on a global scale [9,10].

Historically, lignin was predominantly seen as a challenge to industries that sought to extract cellulose and hemicellulose from plant biomass [11]. Its robust and complex structure, interwoven with aromatic rings and irregular linkages, posed obstacles to facile processing [12]. Consequently, lignin was relegated to a secondary role, often incinerated or relegated to lower-value applications [13]. However, the paradigm surrounding lignin has been rapidly evolving, and researchers are now recognizing its immense potential to address environmental challenges [14]. Lignin’s resurgence is underscored by its ubiquity in nature and its pivotal role in carbon cycling within ecosystems [15]. As a fundamental component of plant cell walls, lignin contributes to the mechanical support and structural integrity of terrestrial vegetation [16]. Its degradation, orchestrated by a consortium of microorganisms, facilitates the recycling of carbon back into the soil, perpetuating the delicate balance of nutrient cycling. This inherent capacity of lignin to undergo biodegradation forms the basis for its potential as an environmentally benign material for diverse applications, including environmental remediation [17]. Indeed, lignin’s unique properties make it an attractive candidate for a variety of applications, particularly in the realm of environmental remediation [18]. As a polyphenolic compound, lignin possesses a high density of functional groups that lend themselves to interactions with a broad spectrum of pollutants [19]. The development of lignin nanoparticles, harnessing the power of nanotechnology, has further expanded its application potential [20]. These nanoparticles, with their high surface area to volume ratio and enhanced reactivity, hold promise for revolutionizing the way we approach environmental cleanup [21].

Research into lignin nanoparticles’ environmental applications has gained momentum across diverse fields. In the realm of water purification, these nanoparticles have exhibited remarkable adsorption capabilities for heavy metals, organic contaminants, and even emerging pollutants such as pharmaceuticals and personal care products (Sharma et al., 2015). It is reported that several types of nanomaterials have been designed to remove heavy metals using absorption technology, such as metal organic-frameworks, metal oxides nanorods, and polymer functionalized nanocomposites [22]. Certain nanoparticle such as UiO-66-NH2 selectively remove hydrophilic dyes in aqueous media [23]. The functional groups present on lignin’s surface enable strong binding interactions, effectively sequestering contaminants and preventing their dissemination within aquatic ecosystems. This aspect is of critical importance, as the presence of such pollutants in water bodies can have far-reaching ecological and human health implications. Lignin nanoparticles have also demonstrated their potential in soil remediation. Contaminated soils, often resulting from industrial activities or improper waste disposal, pose significant challenges to ecosystem health and agricultural productivity [24]. Here, too, lignin nanoparticles offer a compelling solution. Their ability to adsorb pollutants from soil matrices, coupled with their potential to facilitate bioremediation processes, underscores their versatility [25]. Furthermore, the incorporation of lignin nanoparticles into soil amendments can enhance soil structure and nutrient retention, contributing to sustainable agricultural practices (Liu & Lawton, 2019).

The synthesis of lignin nanoparticles represents a critical juncture in realizing their environmental potential. Researchers have explored various methods to produce nanoparticles with tailored properties, including precipitation, emulsion techniques, and enzymatic modification (Zhang et al., 2019). Each approach imparts distinct characteristics to the nanoparticles, influencing their adsorption capacities, surface chemistry, and compatibility with remediation matrices. The choice of lignin source, whether hardwood, softwood, or agricultural residues, further contributes to the diversity of nanoparticles available for environmental applications [26]. However, the integration of lignin nanoparticles into existing remediation strategies is not without challenges [27]. While their potential is undeniable, their successful deployment necessitates a comprehensive understanding of their interactions with both target pollutants and non-target organisms [28]. Ecotoxicological assessments are imperative to evaluate potential risks and unintended consequences associated with nanoparticle introduction into natural systems. Furthermore, questions of nanoparticle stability, long-term efficacy, and scalability must be addressed to ensure the practicality and sustainability of their implementation [29]. As we embark on this exploration of lignin nanoparticles for environmental remediation, it becomes evident that a multidisciplinary approach is essential. Collaboration among chemists, materials scientists, environmental engineers, and ecologists is crucial to fully unlock the potential of lignin nanoparticles and navigate the challenges that lie ahead. The integration of cutting-edge research findings with real-world applications is vital to translate laboratory successes into impactful solutions that address the complex and interconnected challenges of environmental degradation [30].

This review aims to provide an extensive overview of the emerging field of lignin nanoparticles in environmental remediation. By delving into their synthesis, unique properties, applications, challenges, and future prospects, this comprehensive examination seeks to shed light on the transformative potential of lignin nanoparticles. As the world seeks sustainable remedies to the environmental quandaries that beset it, lignin nanoparticles stand as a beacon of hope, offering the possibility of a cleaner, healthier, and more harmonious coexistence between humanity and nature.

## 2. Lignin Nanoparticles: Synthesis and Tailoring for Environmental Applications

The synthesis and tailoring of lignin nanoparticles hold paramount importance in realizing their potential for environmental remediation. A range of techniques has been explored to produce nanoparticles with customized properties suitable for diverse applications. Precipitation methods involve controlled solvent evaporation, leading to the formation of nanoparticles with tunable sizes and morphologies [31]. The synthesis and tailoring of lignin nanoparticles (LNPs) are critical for their effective application in environmental remediation [32]. Among the various techniques for nanoparticle production, precipitation methods stand out due to their ability to produce nanoparticles with precise control over size, morphology, and surface characteristics [33]. This section delves into the precipitation methods used in the synthesis of LNPs, emphasizing their mechanisms, advantages, and the ability to tailor properties for specific applications.

### 2.1. Precipitation Techniques

#### 2.1.1. Controlled Solvent Evaporation

Controlled solvent evaporation is a widely used precipitation technique where lignin is dissolved in a solvent, and nanoparticles are formed as the solvent gradually evaporates [34]. This method allows for the manipulation of particle size and morphology through careful control of evaporation rates and conditions. In this technique, lignin is initially dissolved in a volatile solvent to form a homogeneous solution [35]. Upon evaporation of the solvent, lignin molecules become concentrated, leading to nucleation and growth of nanoparticles. The rate of solvent evaporation can be controlled to influence the size and shape of the resulting nanoparticles. This method is relatively simple and cost-effective [36]. It also offers flexibility in controlling nanoparticle size and morphology by adjusting parameters such as solvent choice, evaporation rate, and temperature [37].

#### 2.1.2. Anti-Solvent Precipitation

Anti-solvent precipitation involves the addition of a non-solvent or anti-solvent to a lignin solution, which decreases the solubility of lignin and induces nanoparticle formation [37,38]. This method is effective in producing nanoparticles with controlled sizes and morphologies. Lignin is first dissolved in a suitable solvent. When an anti-solvent is added, it reduces the solubility of lignin, causing it to precipitate out of the solution [39]. The properties of the nanoparticles can be tailored by adjusting the type and amount of anti-solvent and the rate of its addition [40]. This technique allows for precise control over particle size and distribution by varying the anti-solvent concentration and addition rate. It is also useful for producing nanoparticles with narrow size distributions.

#### 2.1.3. Electrostatic Precipitation

Electrostatic precipitation uses electrostatic forces to induce the formation of lignin nanoparticles. This method leverages the interaction between charged particles to control nanoparticle formation and growth. Lignin is dissolved in a solution where it is then subjected to an electric field [41]. The electrostatic forces cause lignin molecules to aggregate and form nanoparticles. The size and distribution of nanoparticles can be controlled by adjusting the strength of the electric field and the concentration of lignin This method allows for precise control over nanoparticle size and can be scaled up for industrial applications. It also provides the ability to produce nanoparticles with specific surface charges, which can be beneficial for targeted environmental remediation [42].

### 2.2. Emulsion Techniques for Lignin Nanoparticle Synthesis and Targeted Pollutant Removal

Emulsion techniques, on the other hand, allow for the creation of stable emulsions where lignin can encapsulate hydrophobic pollutants, presenting a novel approach for targeted pollutant removal [41]. Emulsion techniques are emerging as effective methods for synthesizing lignin nanoparticles (LNPs) with the ability to encapsulate hydrophobic pollutants. These methods leverage the formation of stable emulsions to incorporate lignin into nanoparticles that can interact with and remove pollutants from various environments [43]. This section explores the principles, advantages, and applications of emulsion techniques in the context of lignin nanoparticle synthesis, emphasizing their potential for targeted pollutant removal.

#### 2.2.1. Oil-in-Water (O/W) Emulsions

In oil-in-water (O/W) emulsions, lignin is dissolved in an organic solvent that forms the oil phase, while water acts as the continuous phase. The emulsification process involves mixing the oil phase with the aqueous phase under shear force, resulting in the formation of small droplets of the oil phase dispersed in water [44]. Upon solvent evaporation or solidification, lignin nanoparticles are formed within the droplets. O/W emulsions are particularly advantageous for encapsulating hydrophobic pollutants within lignin nanoparticles. This method allows for precise control over particle size and morphology by adjusting parameters such as the concentration of lignin, the type of surfactant, and the mixing conditions [45].

#### 2.2.2. Water-in-Oil (W/O) Emulsions

In water-in-oil (W/O) emulsions, water serves as the dispersed phase, while the organic solvent forms the continuous oil phase. Lignin is dissolved in the oil phase, and when the water phase is introduced, it forms droplets dispersed within the oil phase [46,47]. This method is useful for creating lignin nanoparticles where the water droplets can be used to carry and encapsulate hydrophobic pollutants. W/O emulsions are beneficial for forming nanoparticles with unique structural properties, such as core-shell configurations, where lignin forms the shell around the water droplets. This approach enhances the encapsulation of hydrophobic pollutants and provides a controlled release mechanism [48].

#### 2.2.3. Reverse Emulsion Polymerization

Reverse emulsion polymerization involves the formation of an emulsion where the aqueous phase is dispersed in an organic solvent. Lignin is incorporated into the aqueous phase, and upon polymerization, nanoparticles are formed within the droplets of the aqueous phase [49]. This technique can be used to produce lignin nanoparticles with specific surface functionalities and encapsulation capabilities [50]. This method offers high control over the size and functionality of the nanoparticles. The polymerization process within the emulsion droplets allows for the creation of lignin nanoparticles with tailored properties for enhanced pollutant removal [51].

#### 2.2.4. Solvent Evaporation from Emulsions

In this method, lignin is dissolved in an organic solvent and mixed with an aqueous phase to form an emulsion. The solvent is then gradually evaporated, causing lignin to precipitate and form nanoparticles. This process can be fine-tuned to control the size and morphology of the nanoparticles by adjusting the rate of solvent evaporation and the emulsion composition [52]. Solvent evaporation from emulsions allows for the production of lignin nanoparticles with well-defined sizes and structures. It is particularly useful for encapsulating hydrophobic pollutants within the nanoparticles during the precipitation process [43].

### 2.3. Enzymatic Modification Techniques

#### 2.3.1. Laccase-Mediated Oxidation

Laccase, a copper-containing enzyme, catalyzes the oxidative polymerization of lignin phenolic units, leading to the formation of new cross-linked structures. This modification introduces additional functional groups and alters the lignin’s surface properties, enhancing its interaction with various contaminants [53]. Laccase-mediated oxidation allows for the introduction of diverse functional groups, such as carboxyl and methoxy groups, which can improve the lignin’s affinity for different pollutants. The process is generally mild and environmentally friendly compared to chemical modifications [54].

#### 2.3.2. Peroxidase-Catalyzed Modification

Peroxidases, such as manganese peroxidase (MnP) and lignin peroxidase (LiP), catalyze the breakdown of lignin’s aromatic structures, leading to the formation of various functionalized degradation products [55]. These enzymes facilitate the introduction of reactive groups like aldehydes and carboxyls, which can enhance the lignin’s interactions with contaminants [56]. The peroxidase-catalyzed approach enables the selective modification of lignin, creating materials with high reactivity towards specific pollutants [57]. This method is also capable of degrading complex lignin structures, making it suitable for producing highly functionalized lignin derivatives.

#### 2.3.3. Ligninase Treatment

Ligninase enzymes specifically target the breakdown of lignin’s complex structures, leading to modifications that enhance surface functionalities [58]. These enzymes cleave the ether bonds in lignin, resulting in smaller lignin fragments with increased surface reactivity. Ligninase treatment provides a high degree of control over the structural modifications of lignin, resulting in materials with tailored surface properties [59]. This method is particularly useful for preparing lignin-based materials with enhanced interactions for specific applications.

#### 2.3.4. Cellulase-Catalyzed Modification

Cellulases, although primarily used for cellulose degradation, can also influence lignin structure. By cleaving cellulose-lignin bonds, cellulases facilitate the exposure of lignin’s internal functional groups and modify its surface properties. This method can enhance the accessibility of functional groups within lignin, improving its interactions with contaminants. It also contributes to the formation of lignin materials with modified surface characteristics.

Enzymatic modifications of lignin provide a high degree of control over its structure and properties, enabling the tailoring of surface functionalities for enhanced interactions with specific contaminants [60]. Each synthesis method offers distinct advantages for particular environmental challenges. Precipitation techniques allow for the production of nanoparticles with well-defined sizes, which is particularly advantageous for adsorption-based remediation strategies [61]. Emulsion methods enable the creation of lignin-based carriers for pollutants, facilitating controlled and targeted pollutant release [62]. Enzymatic modifications provide a versatile platform for surface engineering, enabling the tailoring of nanoparticles’ chemical properties to optimize interactions with specific contaminants [63].

Additionally, the choice of lignin source significantly influences the properties of the resulting nanoparticles [64]. Hardwood lignin, rich in polyphenolic content, has been shown to exhibit excellent adsorption capabilities for heavy metals due to its abundance of phenolic hydroxyl groups [65]. Softwood lignin, with its intricate and complex structure, can lead to the production of nanoparticles with enhanced mechanical properties, making them suitable for applications requiring structural integrity [66]. Agricultural residues as a lignin source contribute to sustainable practices, aligning with the principles of circular economy and waste utilization [67]. In conclusion, the synthesis and tailoring of lignin nanoparticles present a versatile toolkit for designing effective solutions for environmental remediation. The careful selection of synthesis techniques and lignin sources enables the creation of nanoparticles optimized for specific environmental challenges, thereby unlocking the transformative potential of lignin in environmental applications.

## 3. Unique Properties of Lignin Nanoparticles for Environmental Remediation

**Figure 1:**
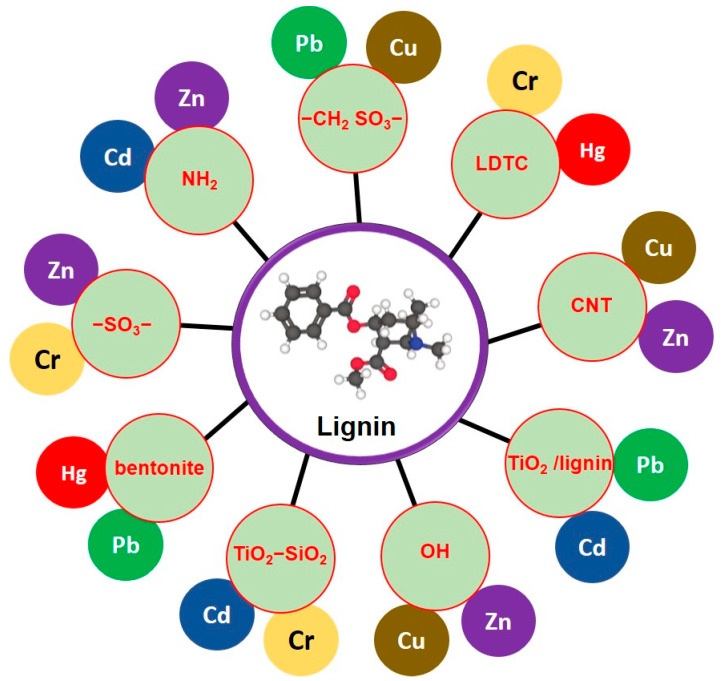
Modified lignin-based adsorbents and their role in heavy metal absorption. (CNT) carbon nanotubes, (TiO_2_) titanium dioxide, (SiO_2_) silicon dioxide, (NH_2_) amino group, (SO_3_) sulfur trioxide, (OH) hydroxyl group, (LDTC) lignin dithiocarbamate, and (CH_2_SO_3_) methanesulfonic acid. This figure is developed from the review article outlined by Ge et al. [65], American Chemical Society, 2018.

Lignin nanoparticles possess a distinctive set of properties that make them highly attractive candidates for environmental remediation applications [68]. Their remarkable attributes stem from both the inherent nature of lignin and the nanoscale morphology achieved through various synthesis methods [69]. Polyphenolic compounds, characterized by the presence of multiple phenolic (hydroxyl) groups on their molecular structures, have gained significant attention due to their remarkable adsorption capabilities in various environmental and industrial applications (Figure 1) [70]. The unique chemical properties of polyphenols make them effective adsorbents for a wide range of pollutants, including heavy metals, organic dyes, and organic contaminants [71]. The adsorption capacity of polyphenolic compounds can be attributed to their functional groups, such as hydroxyl (-OH), carboxyl (-COOH), and phenolic (-C6H5OH) groups [72]. These functional groups provide numerous active sites for chemical interactions, leading to strong adsorption forces [72]. Studies have shown that the number and arrangement of these functional groups on polyphenolic molecules greatly influence their adsorption performance (Figure 1) [73]. The adsorption of heavy metals by tannins, a subclass of polyphenolic compounds abundant in plant materials [74], was investigated. They found that the multiple hydroxyl groups present in tannins chelated with metal ions, forming stable complexes and effectively removing heavy metals from aqueous solutions [75]. This demonstrates the importance of hydroxyl groups in the adsorption of metal contaminants.

Furthermore, carboxylic groups in polyphenolic compounds were found to enhance the adsorption of organic pollutants [76]. Researchers studied the adsorption of organic dyes by graphene oxide functionalized with polyphenolic compounds containing carboxyl groups. The presence of carboxyl groups on the polyphenolic molecules facilitated electrostatic interactions and π-π stacking interactions with the dye molecules, leading to improved adsorption efficiency [77]. In addition to hydroxyl and carboxyl groups, the phenolic groups in polyphenols play a crucial role in adsorption processes [78]. Phenolic groups have strong electron-donating abilities and can form hydrogen bonds with various contaminants. This interaction was demonstrated by Wu, Du et al. 2020 in their study on the adsorption of phenolic compounds from aqueous solutions using activated carbon modified with polyphenolic materials [79]. The phenolic groups on the adsorbent surface promoted the adsorption of phenolic compounds through hydrogen bonding. Therefore, polyphenolic compounds, with their rich diversity of functional groups, offer versatile adsorption capabilities for a wide range of contaminants in environmental and industrial applications. The hydroxyl, carboxyl, and phenolic groups present in polyphenols provide a myriad of active sites for adsorption interactions, making them promising materials for the development of effective adsorbents.

Lignin nanoparticles, derived from lignocellulosic biomass, have garnered significant attention in recent years due to their exceptional properties, including a high surface area to volume ratio and enhanced reactivity [80]. These characteristics make lignin nanoparticles promising candidates for a wide range of applications, particularly in environmental remediation and sustainable materials development [81]. Lignin, as a complex natural polymer, is typically found in plant cell walls in a bulk form [82]. However, through various processing techniques such as acid hydrolysis, mechanical treatment, and enzymatic methods, lignin can be transformed into nanoparticles with substantially reduced dimensions compared to bulk lignin [83]. This nanoscale size results in a remarkably high surface area to volume ratio for lignin nanoparticles [84]. The increased surface area arises from the unique structure of lignin nanoparticles, which is characterized by a porous and interconnected network of aromatic phenolic compounds [85]. This intricate network provides a multitude of active sites on the nanoparticle’s surface, making them highly accessible for adsorption interactions [86]. The high surface area to volume ratio of lignin nanoparticles contributes significantly to their enhanced reactivity [87]. These nanoparticles offer an increased number of active sites for chemical interactions with target molecules, such as pollutants or organic compounds [71]. The active sites primarily consist of hydroxyl (-OH) and phenolic groups (-C6H5OH) present on the surface of lignin nanoparticles [88].

Hydroxyl and phenolic groups are known to form hydrogen bonds and other chemical interactions with various contaminants. This results in improved adsorption efficiency and selectivity [89]. Furthermore, the nanoscale dimensions of lignin nanoparticles allow for better dispersion in solution, increasing the probability of contact with target molecules and promoting rapid adsorption kinetics [90]. Additionally, the porous structure of lignin nanoparticles, with mesopores and micropores, provides ample space for the accommodation of molecules of different sizes [91]. This feature enhances their versatility as adsorbents, allowing them to effectively remove a wide range of pollutants from various matrices [92]. Lignin nanoparticles, owing to their unique structural characteristics and functional groups, have demonstrated remarkable compatibility with a wide range of pollutants in various environmental remediation and adsorption applications [65]. This section explores the diverse pollutants with which lignin nanoparticles exhibit compatibility and the underlying mechanisms of their interactions.

Lignin nanoparticles have shown significant promise in the removal of heavy metal pollutants from aqueous environments [93]. The hydroxyl (-OH) and phenolic (-C6H5OH) groups on the surface of lignin nanoparticles enable complexation and chelation with heavy metal ions [94]. These interactions form stable complexes, reducing the concentration of heavy metals in solution. This compatibility with heavy metal pollutants makes lignin nanoparticles a valuable adsorbent for water and soil remediation in contaminated areas [94]. In wastewater treatment, the removal of organic dyes is a critical challenge. Lignin nanoparticles have demonstrated excellent compatibility with various organic dyes due to their electrostatic interactions, π-π stacking, and hydrogen bonding capabilities [95]. These interactions result in efficient adsorption of organic dyes onto lignin nanoparticle surfaces, leading to effective color removal from industrial effluents [96]. Lignin nanoparticles have also shown compatibility with a wide range of organic contaminants, including pesticides, pharmaceuticals, and volatile organic compounds (VOCs) [97]. The porous structure of lignin nanoparticles allows for the adsorption of organic molecules of different sizes and properties. The presence of functional groups on lignin nanoparticle surfaces, such as carboxyl (-COOH) and hydroxyl (-OH) groups, facilitates chemical interactions that promote the adsorption of organic contaminants [64,98]. In addition to water-soluble pollutants, lignin nanoparticles exhibit compatibility with hydrophobic substances, such as oil and hydrophobic organic compounds [99]. Lignin nanoparticles can be functionalized to enhance their hydrophobicity, allowing them to selectively adsorb and remove oil and hydrophobic contaminants from water surfaces [100]. This property has applications in oil spill cleanup and water purification. Beyond pollutant removal, lignin nanoparticles have been explored for their potential in nutrient removal, specifically for phosphate and nitrate ions from water systems [101]. Their compatibility with these nutrients arises from their ability to form ion-exchange complexes through electrostatic interactions [101]. This property has implications for managing nutrient pollution in aquatic ecosystems.

## 4. Application of Lignin Nanoparticles in Water Purification

**Figure 2:**
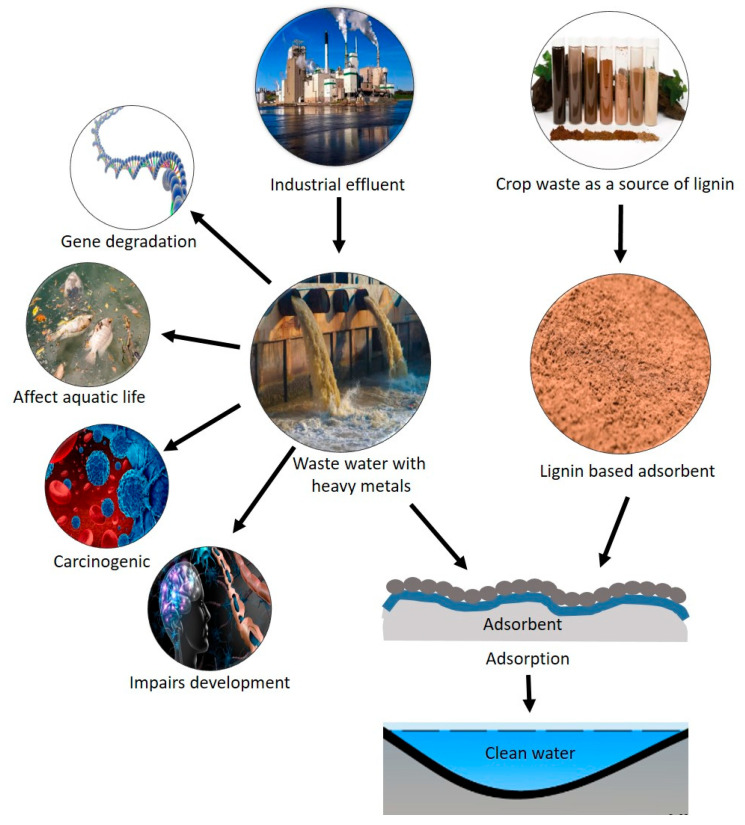
Schematic representation of the water-purification process.

Water pollution is a growing global concern that demands innovative and sustainable solutions for effective water purification. Lignin, a complex polyphenolic compound abundant in the cell walls of plants, is garnering attention as a potential candidate for environmental remediation due to its abundant availability, biodegradability, and unique physicochemical properties [102]. Lignin nanoparticles, derived from lignocellulosic biomass through controlled processes, have shown promising applications in various aspects of water purification (Figure 2) [103]. This literature review delves into the potential applications of lignin nanoparticles in water purification, highlighting their capacity for heavy metal adsorption, removal of organic contaminants, mitigation of emerging pollutants, and implications for aquatic ecosystem health.

One of the critical environmental challenges worldwide is the contamination of water sources with heavy metals such as lead (Pb), cadmium (Cd), mercury (Hg), and chromium (Cr) [104]. These heavy metals are toxic and pose significant health risks to both humans and aquatic ecosystems. Around 75% of the elements in the periodic table are metals, but only a few are essential for life. Non-essential metals like cadmium, lead, mercury, arsenic, and aluminum have no biological function and can be harmful even in small amounts. Essential trace elements like copper, chromium, zinc, and iron are crucial for biological processes but become toxic at high concentrations [105]. Heavy metal in plant-based food are absorbed by the gastrointestinal tract and transferred into the human body [106].

Adsorption processes involving lignin nanoparticles have emerged as an effective and sustainable solution for the removal of heavy metals from water [107]. Lignin nanoparticles possess a high surface area to volume ratio and a wealth of functional groups, including hydroxyl (-OH), phenolic (-C6H5OH), and carboxyl (-COOH) groups, on their surface [108]. These functional groups play a pivotal role in the adsorption of heavy metals [109]. When heavy metal ions are present in water, they undergo interactions with these functional groups on the surface of lignin nanoparticles [110]. The interactions between heavy metal ions and lignin nanoparticles primarily involve complexation and chelation processes [65]. Complexation occurs when heavy metal ions are coordinated and bound to the hydroxyl and carboxyl groups present on the lignin nanoparticle surface [111]. This interaction forms stable complexes that effectively reduce the concentration of heavy metals in the aqueous phase [112]. Chelation, on the other hand, involves the formation of multi-ring structures where heavy metal ions are trapped within the lignin nanoparticle structure, often involving the phenolic groups [113]. This phenomenon enhances the removal efficiency and stability of heavy metal ions in the adsorption process [114]

The high surface area to volume ratio of lignin nanoparticles, combined with the multitude of active sites offered by their functional groups, results in a substantial adsorption capacity for heavy metals [65]. Studies have demonstrated impressive adsorption efficiencies for heavy metal removal using lignin nanoparticles as adsorbents [115]. The removal of organic contaminants from water sources is a critical concern for environmental protection and public health [116]. Lignin nanoparticles, derived from lignocellulosic biomass, have emerged as promising adsorbents for the efficient removal of a wide range of organic contaminants [117]. This literature review explores the applications, mechanisms, and advantages of using lignin nanoparticles for organic contaminant removal. With the progressive development of global industrialization and medical technology, better maintenance of human vital signs is imperative. Therefore, developing advanced technology to remove pollutants from the aquatic environment is essential to protect human health and achieve carbon neutralization. Photocatalysts have gained significant attention for their ability to remove harmful pollutants from water, thanks to their advantages of being non-toxic, cost-effective, highly stable, and energy-efficient [118]. Selecting an appropriate semiconductor photocatalyst is crucial. The photocatalyst should have a suitable band edge to produce free radicals, demonstrate strong visible light responsiveness, and absorb enough visible light to effectively facilitate the catalytic degradation of pollutants [118]. Bi_2_MoO_6_ is a photocatalyst known for its excellent performance, thanks to its unique layered structure and good thermal stability. Its general formula is Bi_2_O_3_·nMoO_3_, where the different forms are α-Bi_2_Mo_3_O_12_, β-Bi_2_Mo_2_O_9_, and γ-Bi_2_MoO_6_. Of these, γ-Bi_2_MoO_6_ stands out because it effectively responds to visible light and is a new type of bismuth-based material used in photocatalysis. This makes γ-Bi_2_MoO_6_ very promising for applications in breaking down pollutants using light [119,120]. Likewise, a photocatalyst combining g-C_3_N_4_ nanosheets with Bi_4_Ti_3_O_12_ nanofibers is made using a method that involves electrospinning and self-assembly. The Bi_4_Ti_3_O_12_/g-C_3_N_4_ photocatalyst shows a notable improvement in producing hydrogen peroxide (H_2_O_2_), with a yield of 1650 μmol∙g⁻^1^∙h⁻^1^, and can efficiently generate H_2_O_2_ directly from pure water [121]. It has also been reported that NiS@Ta_2_O_5_ core-shell nanofibers are used to efficiently convert CO_2_ into hydrocarbons [122]. The key function of NiS@Ta_2_O_5_ is to enhance this conversion by separating and extracting charges more effectively through an S-scheme heterojunction, which improves the photocatalytic process [122].

Lignin nanoparticles have shown remarkable versatility in adsorbing various organic contaminants, including pesticides, pharmaceuticals, dyes, and volatile organic compounds (VOCs) [123]. Their porous structure, high surface area, and abundant functional groups enable effective interactions with organic molecules [124]. This versatility positions lignin nanoparticles as valuable materials for addressing diverse organic pollutant challenges. The success of lignin nanoparticles in organic contaminant removal can be attributed to the functional groups present on their surface, such as hydroxyl (-OH), phenolic (-C6H5OH), and carboxyl (-COOH) groups [125]. These functional groups engage in various adsorption mechanisms, including:

- Electrostatic Interaction: Lignin nanoparticles can interact with charged organic contaminants through electrostatic attraction. Negatively charged functional groups on lignin nanoparticles can attract positively charged organic molecules (Wang et al., 2020).
- π-π Stacking: Aromatic organic contaminants often undergo π-π stacking interactions with lignin nanoparticles, facilitated by the presence of phenolic rings in lignin structures (Shen et al., 2017).
  Hydrogen Bonding: The hydroxyl and carbonyl groups on lignin nanoparticle surfaces can form hydrogen bonds with organic compounds, promoting their adsorption [124].
- Pore Entrapment: The porous structure of lignin nanoparticles allows for the physical trapping and immobilization of organic molecules within their pores [124].

The combination of their structural characteristics and surface chemistry results in a high adsorption capacity for lignin nanoparticles when removing organic contaminants. Studies have demonstrated impressive removal efficiencies for various organic pollutants, highlighting the efficacy of lignin nanoparticles (Shen et al., 2017; Wang et al., 2020). Lignin, as a natural polymer, offers the advantage of environmental sustainability. It is renewable, biodegradable, and non-toxic, making lignin nanoparticles an eco-friendly alternative to synthetic adsorbents [126].

The emergence of novel pollutants like microplastics and nanoparticles has heightened the need for advanced water treatment methods. Lignin nanoparticles can play a role in removing these pollutants through adsorption and coagulation mechanisms. Their potential to form aggregates with microplastics and nanoparticles aids in their removal from water matrices [127]. Contaminant accumulation disrupts aquatic ecosystems, leading to bioaccumulation and ecological imbalances [128]. By effectively removing pollutants, lignin nanoparticles contribute to the restoration of ecosystem health. Additionally, the low toxicity of lignin nanoparticles to aquatic organisms further supports their application in water purification without causing additional harm [26].

## 5. Utilizing Lignin Nanoparticles for Soil Remediation

**Figure 3:**
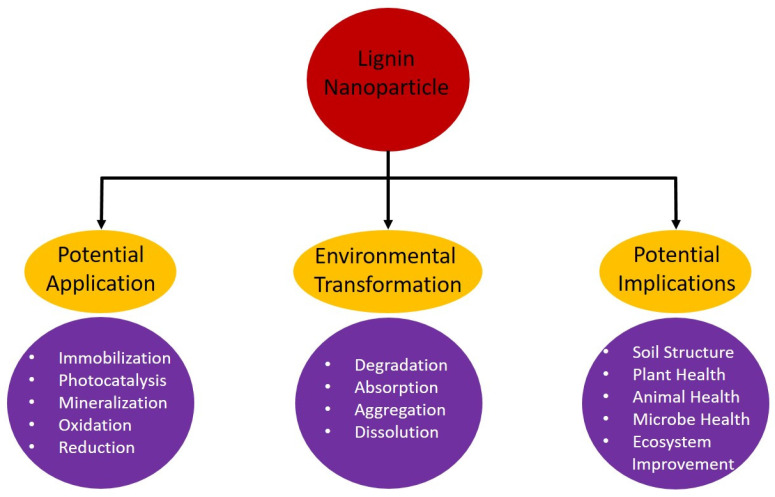
Illustration depicting the roles of lignin nanoparticles in the soil-remediation process. This figure is reported in a review article by Qian et al., 2022 [139].

Soil contamination is a pervasive and pressing environmental concern that jeopardizes ecosystems and human well-being [129]. In the quest for sustainable and efficient soil-remediation techniques, lignin nanoparticles have emerged as a promising candidate (Figure 3). Derived from lignocellulosic biomass, these nanoscale materials exhibit a unique set of properties that render them highly effective in addressing soil pollution [130]. Lignin nanoparticles possess exceptional adsorption capabilities due to their significant surface area and the presence of diverse functional groups, including hydroxyl and phenolic moieties [65]. These attributes enable them to effectively sequester a wide spectrum of contaminants, including heavy metals, organic pollutants, and dyes [131]. One of the key advantages of lignin nanoparticles is their environmental sustainability [132]. Being derived from renewable sources and biodegradable, they align with the principles of green remediation [133]. Moreover, lignin nanoparticles can be obtained as byproducts from the pulp and paper industry, making them a cost-effective and eco-friendly option for soil remediation [132].

Beyond their contaminant-removal capacity, lignin nanoparticles contribute to enhancing soil health (Figure 3) [134]. They improve soil structure by promoting aggregation and moisture retention, and they serve as a carbon source for beneficial microorganisms, stimulating microbial activity and nutrient cycling, which are vital for sustainable land use and ecological restoration [135]. While lignin nanoparticles hold immense potential, challenges exist in scaling up their production and ensuring their safe deployment in field applications [136]. Future research endeavors should focus on optimizing their properties, evaluating long-term environmental impacts, and devising practical strategies for their integration into soil-remediation practices [137]. So, lignin nanoparticles represent a sustainable and versatile solution for soil remediation [126]. Their remarkable adsorption capabilities, environmental friendliness, and potential for improving soil quality make them a valuable asset in the ongoing effort to mitigate soil contamination and safeguard environmental health [138].

## 6. Challenges in Deploying Lignin Nanoparticles for Environmental Remediation

The utilization of lignin nanoparticles in environmental remediation holds immense promise, but their practical implementation faces several challenges that must be addressed to ensure their efficacy and sustainability [140]. This literature review delves into the critical challenges associated with deploying lignin nanoparticles for environmental remediation, focusing on eco-toxicological assessments and risk evaluation, nanoparticle stability and long-term efficacy, and the scalability and practical implementation of these innovative solutions [141]. Before the widespread use of lignin nanoparticles in environmental remediation, thorough ecotoxicological assessments are imperative [142]. While lignin is generally considered biocompatible, its nanoparticles’ novel characteristics warrant a comprehensive understanding of their interactions with various organisms and ecosystems [143]. Studying potential toxicity, bioaccumulation, and unintended ecological consequences is crucial to avoid inadvertently causing harm while attempting to remediate contamination [144]. Proper risk evaluation frameworks need to be developed to assess the potential hazards and benefits associated with their application [145].

The stability of lignin nanoparticles in various environmental conditions is essential for sustained remediation efforts [146]. Factors such as pH, temperature, and the presence of competing ions can influence nanoparticle aggregation and degradation rates [147]. Ensuring long-term efficacy requires optimizing nanoparticle formulation and surface modification to enhance their stability, preventing premature loss of adsorption capacity and effectiveness [148]. Moreover, the potential release of nanoparticles from treated matrices into the environment must be carefully evaluated to prevent unintended secondary contamination [149]. While laboratory-scale studies demonstrate the potential of lignin nanoparticles, translating these findings to real-world scenarios presents significant challenges [150]. Achieving the necessary scale for effective environmental cleanup demands cost-effective production methods and large-scale nanoparticle synthesis techniques [151]. Moreover, integrating lignin nanoparticle-based solutions into existing water treatment or soil-remediation infrastructure requires careful consideration of compatibility, practicality, and regulatory compliance [152]. Collaborative efforts among researchers, engineers, and policymakers are essential to ensure the seamless integration of these innovations into environmental management practices [153].

## 7. Multidisciplinary Approach to Harnessing Lignin Nanoparticles

Harnessing the potential of lignin nanoparticles for environmental remediation requires a multidisciplinary approach that bridges the gap between laboratory research and real-world applications [154]. Collaborations among chemists, materials scientists, engineers, and ecologists are crucial for addressing the complex challenges of environmental degradation and developing innovative solutions [155]. This literature review explores the importance of multidisciplinary approaches in utilizing lignin nanoparticles, focusing on collaboration, bridging research with applications, and addressing the multifaceted challenges posed by environmental degradation.

**Figure 4:**
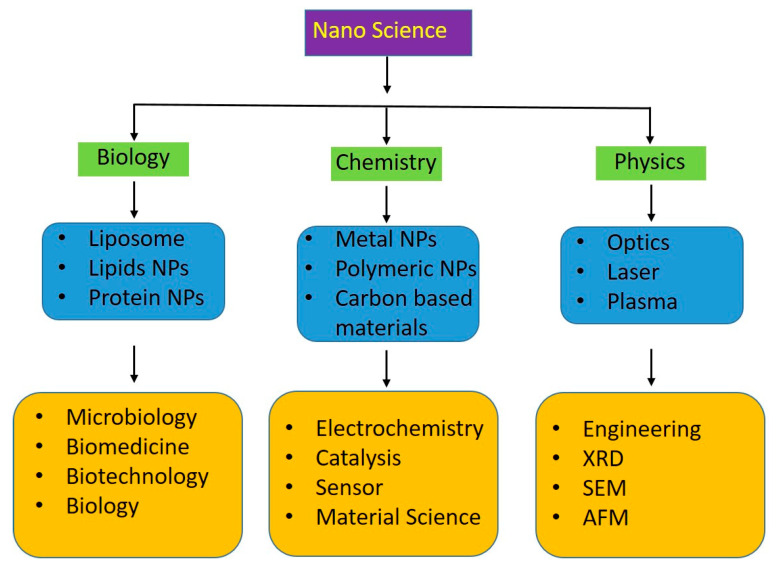
Visual representation of a multidisciplinary team collaborating to harness lignin nanoparticles for environmental remediation, addressing diverse aspects from nanoparticle design to real-world applications.

The complexity of environmental issues demands expertise from various fields [156]. Collaboration among chemists, materials scientists, engineers, and ecologists is essential for designing lignin nanoparticles with optimized properties for environmental remediation shown in Figure 4 [157]. Chemists can tailor nanoparticle characteristics for specific applications, materials scientists can ensure stability and compatibility, engineers can develop scalable production methods, and ecologists can assess ecological impacts [157]. These interdisciplinary teams bring diverse perspectives that enhance the effectiveness and safety of lignin nanoparticle solutions [158]. While promising findings emerge from laboratory experiments, translating these into practical solutions requires an understanding of real-world complexities [159]. Multidisciplinary teams can bridge this gap by designing experiments that mimic field conditions and by considering economic, regulatory, and logistical factors [160]. Collaborative efforts can develop prototypes and pilot studies that test the feasibility of lignin nanoparticle applications in real environments, providing valuable insights for refining their design and implementation.

Environmental degradation is a multifaceted challenge that often involves intricate interactions among pollutants, ecosystems, and human activities [161]. Multidisciplinary teams can address these complexities by developing holistic solutions [161]. For instance, a collaborative effort can integrate lignin nanoparticles into a larger framework that combines physical, chemical, and biological approaches for remediation [162]. This comprehensive strategy addresses not only contaminant removal but also ecosystem restoration and long-term sustainability [163]. Collaborations among chemists, materials scientists, engineers, and ecologists enable the design, testing, and implementation of lignin nanoparticle-based solutions that are effective, safe, and environmentally conscious. By bridging the gap between laboratory research and practical applications, multidisciplinary teams can play a pivotal role in developing innovative strategies to combat environmental degradation and promote a more sustainable future.

## 8. Future Prospects and Implications of Lignin Nanoparticles in Sustainable Remediation

The application of lignin nanoparticles in environmental remediation holds the promise of transforming traditional cleanup methods into more sustainable and efficient strategies. This literature review explores the potential transformative impact, advancement of circular and sustainable paradigms, and the coexistence between humanity and nature through the integration of lignin nanoparticle solutions. Emphasizing these future prospects, this review examines the implications of lignin nanoparticles in shaping the landscape of environmental remediation strategies.

### 8.1. Potential Transformative Impact on Environmental Cleanup

Lignin nanoparticles have the potential to revolutionize environmental cleanup by providing innovative solutions for addressing diverse pollution challenges [164]. Their versatile applications, ranging from heavy metal removal to organic pollutant degradation, offer a comprehensive toolkit for remediating contaminated water and soil [165]. As research continues and applications are refined, the integration of lignin nanoparticles into existing systems could lead to faster, more efficient, and more sustainable approaches to environmental cleanup [166].

### 8.2. Advancing Circular and Sustainable Paradigms

Lignin, a renewable and abundant resource, offers the advantage of aligning with circular and sustainable principles [167]. By utilizing lignocellulosic waste as a precursor for nanoparticle synthesis, lignin nanoparticles contribute to reducing waste and promoting a circular economy [168]. Their biodegradability and potential for enhancing soil health further underscore their compatibility with sustainable practices [169]. Lignin nanoparticles exemplify how leveraging natural resources can drive advancements in environmental remediation while minimizing ecological footprints [170].

### 8.3. Coexistence between Humanity and Nature through Nanoparticles Solutions

The relationship between human activities and the environment has often been one of conflict, but lignin nanoparticles offer a bridge to coexistence [171]. As these nanoparticles are designed to target and mitigate pollution, they can aid in restoring ecosystems and safeguarding water and soil quality [172]. The implementation of lignin nanoparticle-based solutions promotes a harmonious relationship between human development and environmental preservation, illustrating how scientific innovation can be harnessed for the benefit of both humanity and nature [146].

## 9. Conclusions

In response to escalating environmental challenges, the exploration of lignin nanoparticles offers a promising avenue for sustainable remediation. Once considered an industrial byproduct, lignin has emerged as a key player in promoting carbon cycling and ecological balance. This review highlights the synthesis, properties, and environmental applications of lignin nanoparticles, particularly in addressing water pollution and soil contamination. Despite their potential, challenges such as ecotoxicological risks, stability, and scalability remain. The multidisciplinary collaboration is essential to unlocking their full potential. Ultimately, lignin nanoparticles represent a fusion of scientific innovation and sustainable practices, offering a path toward a cleaner, more harmonious future.

## References

[1] Potts R., Dommain R., Moerman J.W., Behrensmeyer A.K., Deino A.L., Riedl S., Beverly E.J., Brown E.T., Deocampo D., Kinyanjui R. (2020). Increased ecological resource variability during a critical transition in hominin evolution. Sci. Adv..

[2] Aguilera R.V., Aragón-Correa J.A., Marano V., Tashman P.A. (2021). The corporate governance of environmental sustainability: A review and proposal for more integrated research. J. Manag..

[3] Lubchenco J. (1998). Entering the century of the environment: A new social contract for science. Science.

[4] Leonard A. (2010). The Story of Stuff: How Our Obsession with Stuff Is Trashing the Planet, Our Communities, and Our Health-and a Vision for Change.

[5] Becker P. (2014). Sustainability Science: Managing Risk and Resilience for Sustainable Development.

[6] Lucertini G., Musco F. (2020). Circular urban metabolism framework. One Earth.

[7] Meadows D., Randers J. (2012). The Limits to Growth: The 30-Year Update.

[8] Singh S.B. (2021). Emerging sustainable nanomaterials and their applications in catalysis and corrosion control. Curr. Nanosci..

[9] Payne C.M., Knott B.C., Mayes H.B., Hansson H., Himmel M.E., Sandgren M., Stahlberg J., Beckham G.T. (2015). Fungal cellulases. Chem. Rev..

[10] Council N.R. (2009). A New Biology for the 21st Century.

[11] Sharma V.K., Filip J., Zboril R., Varma R.S. (2015). Natural inorganic nanoparticles–formation, fate, and toxicity in the environment. Chem. Soc. Rev..

[12] Yuan J.-C., Huang R., Jiang L.-Y., Liu G.-D., Liu P.-D., Xu W.-R. (2023). Facile production of cellulose nanofibers from raw elephant grass by an aluminum chloride-enhanced acidic deep eutectic solvent. Int. J. Biol. Macromol..

[13] Dale V.H., Kline K.L., Wright L.L., Perlack R.D., Downing M., Graham R.L. (2011). Interactions among bioenergy feedstock choices, landscape dynamics, and land use. Ecol. Appl..

[14] Vasseghian Y., Arunkumar P., Joo S.-W., Gnanasekaran L., Kamyab H., Rajendran S., Balakrishnan D., Chelliapan S., Klemeš J.J. (2022). Metal-organic framework-enabled pesticides are an emerging tool for sustainable cleaner production and environmental hazard reduction. J. Clean. Prod..

[15] Hermann E., Hochuli P.A., Méhay S., Bucher H., Brühwiler T., Ware D., Hautmann M., Roohi G., Yaseen A. (2011). Organic matter and palaeoenvironmental signals during the Early Triassic biotic recovery: The Salt Range and Surghar Range records. Sediment. Geol..

[16] Ragauskas A.J., Beckham G.T., Biddy M.J., Chandra R., Chen F., Davis M.F., Davison B.H., Dixon R.A., Gilna P., Keller M. (2014). Lignin valorization: Improving lignin processing in the biorefinery. Science.

[17] Leadbeater D.R. (2018). Bioprospecting Halotolerant Lignocellulolytic Enzymes from Salt Marsh Ecosystems.

[18] Lizundia E., Sipponen M.H., Greca L.G., Balakshin M., Tardy B.L., Rojas O.J., Puglia D. (2021). Multifunctional lignin-based nanocomposites and nanohybrids. Green Chem..

[19] Mudhoo A., Ramasamy D.L., Bhatnagar A., Usman M., Sillanpää M. (2020). An analysis of the versatility and effectiveness of composts for sequestering heavy metal ions, dyes and xenobiotics from soils and aqueous milieus. Ecotoxicol. Environ. Saf..

[20] Abraham B., Syamnath V., Arun K., Zahra P.F., Anjusha P., Kothakotta A., Chen Y.-H., Ponnusamy V.K., Nisha P. (2023). Lignin-based nanomaterials for food and pharmaceutical applications: Recent trends and future outlook. Sci. Total Environ..

[21] Roy A., Sharma A., Yadav S., Jule L.T., Krishnaraj R. (2021). Nanomaterials for remediation of environmental pollutants. Bioinorg. Chem. Appl..

[22] Zeng J., Qi P., Wang Y., Liu Y., Sui K. (2021). Electrostatic assembly construction of polysaccharide functionalized hybrid membrane for enhanced antimony removal. J. Hazard. Mater..

[23] Fu L., Wang S., Lin G., Zhang L., Liu Q., Fang J., Wei C., Liu G. (2019). Post-functionalization of UiO-66-NH2 by 2, 5-Dimercapto-1, 3, 4-thiadiazole for the high efficient removal of Hg (II) in water. J. Hazard. Mater..

[24] Kim D.Y., Kadam A., Shinde S., Saratale R.G., Patra J., Ghodake G. (2018). Recent developments in nanotechnology transforming the agricultural sector: A transition replete with opportunities. J. Sci. Food Agric..

[25] Matveeva V.G., Bronstein L.M. (2022). From renewable biomass to nanomaterials: Does biomass origin matter?. Prog. Mater. Sci..

[26] Barhoum A., Jeevanandam J., Rastogi A., Samyn P., Boluk Y., Dufresne A., Danquah M.K., Bechelany M. (2020). Plant celluloses, hemicelluloses, lignins, and volatile oils for the synthesis of nanoparticles and nanostructured materials. Nanoscale.

[27] Sethupathy S., Morales G.M., Gao L., Wang H., Yang B., Jiang J., Sun J., Zhu D. (2022). Lignin valorization: Status, challenges and opportunities. Bioresour. Technol..

[28] Bilal M., Ashraf S.S., Barceló D., Iqbal H.M. (2019). Biocatalytic degradation/redefining “removal” fate of pharmaceutically active compounds and antibiotics in the aquatic environment. Sci. Total Environ..

[29] Wiesner M.R., Lowry G.V., Alvarez P., Dionysiou D., Biswas P. (2006). Assessing the risks of manufactured nanomaterials. Environ. Sci. Technol..

[30] Gatto A. (2020). A pluralistic approach to economic and business sustainability: A critical meta-synthesis of foundations, metrics, and evidence of human and local development. Corp. Soc. Responsib. Environ. Manag..

[31] Du B., Li W., Bai Y., Pan Z., Wang Q., Wang X., Ding H., Lv G., Zhou J. (2022). Fabrication of uniform lignin nanoparticles with tunable size for potential wound healing application. Int. J. Biol. Macromol..

[32] Nagar R., Mathur P., Chaturvedi P., Sharma C., Bhatnagar P. (2024). Secondary Metabolites in Green Synthesis of Nanoparticles. Microbial Approaches for Sustainable Green Technologies.

[33] Rao B.G., Mukherjee D., Reddy B.M. (2017). Novel approaches for preparation of nanoparticles. Nanostructures for Novel Therapy.

[34] Azimvand J., Didehban K., Mirshokrai S.A. (2018). Preparation and characterization of lignin polymeric nanoparticles using the green solvent ethylene glycol: Acid precipitation technology. BioResources.

[35] Sameni J., Krigstin S., Sain M. (2017). Solubility of lignin and acetylated lignin in organic solvents. BioResources.

[36] Kumar R., Gupta A., Chawla M., Aadil K.R., Dutt S., Kumar V.B., Chaudhary A. (2020). Advances in nanotechnology based strategies for synthesis of nanoparticles of lignin. Lignin: Biosynthesis and Transformation for Industrial Applications.

[37] Shenashen M.A., El-Safty S.A., Elshehy E.A. (2014). Synthesis, morphological control, and properties of silver nanoparticles in potential applications. Part. Part. Syst. Charact..

[38] Chacon W.D.C., Verruck S., Monteiro A.R., Valencia G.A. (2023). The mechanism, biopolymers and active compounds for the production of nanoparticles by anti-solvent precipitation: A review. Food Res. Int..

[39] Rieland J. (2023). Towards Sustainable Textiles: Microplastics, Coffee, and Closing the Loop. Ph.D. Thesis.

[40] Joye I.J., McClements D.J. (2013). Production of nanoparticles by anti-solvent precipitation for use in food systems. Trends Food Sci. Technol..

[41] Chauhan P.S. (2020). Lignin nanoparticles: Eco-friendly and versatile tool for new era. Bioresour. Technol. Rep..

[42] Zhang Z., Terrasson V., Guénin E. (2021). Lignin nanoparticles and their nanocomposites. Nanomaterials.

[43] Sipponen M.H., Liu L. (2024). Advances in Preparation and Applications of Lignin Nanoparticles. Lignin Chemistry: Characterization, Isolation, and Valorization.

[44] Sekeri S.H.B. (2022). Preparation of a Stabilized Nano-Lignin Emulsifier from Oil Palm Empty Fruit Bunch. Master’s Thesis.

[45] Beckers S. (2020). Controlled Drug Delivery by Lignin Nanocarriers for Sustainable Plant Protection.

[46] Mikkonen K.S. (2020). Strategies for structuring diverse emulsion systems by using wood lignocellulose-derived stabilizers. Green Chem..

[47] Nypelö T.E., Carrillo C.A., Rojas O.J. (2015). Lignin supracolloids synthesized from (W/O) microemulsions: Use in the interfacial stabilization of Pickering systems and organic carriers for silver metal. Soft Matter.

[48] Mudassir M.A., Aslam H.Z., Ansari T.M., Zhang H., Hussain I. (2021). Fundamentals and Design-Led Synthesis of Emulsion-Templated Porous Materials for Environmental Applications. Adv. Sci..

[49] Tang Q., Qian Y., Yang D., Qiu X., Qin Y., Zhou M. (2020). Lignin-based nanoparticles: A review on their preparations and applications. Polymers.

[50] Yusefi M., Chan H., Teow S., Kia P., Lee-Kiun Soon M., Sidik N., Shameli K. (2021). 5-Fluorouracil Encapsulated Chitosan-Cellulose Fiber Bionanocomposites: Synthesis, Characterization and In Vitro Analysis towards Colorectal Cancer Cells. Nanomaterials.

[51] Wang Y.-Y., Meng X., Pu Y., Ragauskas A.J. (2020). Recent advances in the application of functionalized lignin in value-added polymeric materials. Polymers.

[52] O’Donnell P.B., McGinity J.W. (1997). Preparation of microspheres by the solvent evaporation technique. Adv. Drug Deliv. Rev..

[53] Reddy Y.N., Gogde K., Paul S., Bhaumik J. (2021). Lignin to Platform Chemicals and Biomaterials: Chemical and Biological Perspectives. Biomass for Bioenergy and Biomaterials.

[54] Malhotra M., Suman S.K. (2021). Laccase-mediated delignification and detoxification of lignocellulosic biomass: Removing obstacles in energy generation. Environ. Sci. Pollut. Res..

[55] Singh A.K., Bilal M., Iqbal H.M., Raj A. (2021). Lignin peroxidase in focus for catalytic elimination of contaminants—A critical review on recent progress and perspectives. Int. J. Biol. Macromol..

[56] Datta R., Kelkar A., Baraniya D., Molaei A., Moulick A., Meena R.S., Formanek P. (2017). Enzymatic degradation of lignin in soil: A review. Sustainability.

[57] Sellami K., Couvert A., Nasrallah N., Maachi R., Abouseoud M., Amrane A. (2022). Peroxidase enzymes as green catalysts for bioremediation and biotechnological applications: A review. Sci. Total Environ..

[58] Koradiya M. (2024). Advances in Enzymatic Hydrolysis and Saccharification Technology. Biofuels.

[59] Rath S., Pradhan D., Du H., Mohapatra S., Thatoi H. (2024). Transforming lignin into value-added products: Perspectives on lignin chemistry, lignin-based biocomposites, and pathways for augmenting ligninolytic enzyme production. Adv. Compos. Hybrid Mater..

[60] Hu Y., Priya A., Chen C., Liang C., Wang W., Wang Q., Lin C.S.K., Qi W. (2023). Recent advances in substrate-enzyme interactions facilitating efficient biodegradation of lignocellulosic biomass: A review. Int. Biodeterior. Biodegrad..

[61] Di Iorio E., Colombo C., Cheng Z., Capitani G., Mele D., Ventruti G., Angelico R. (2019). Characterization of magnetite nanoparticles synthetized from Fe (II)/nitrate solutions for arsenic removal from water. J. Environ. Chem. Eng..

[62] Ariyanta H.A., Sari F.P., Sohail A., Restu W.K., Septiyanti M., Aryana N., Fatriasari W., Kumar A. (2023). Current roles of lignin for the agroindustry: Applications, challenges, and opportunities. Int. J. Biol. Macromol..

[63] Wang M., Mohanty S.K., Mahendra S. (2019). Nanomaterial-supported enzymes for water purification and monitoring in point-of-use water supply systems. Acc. Chem. Res..

[64] Gao W., Fatehi P. (2019). Lignin for polymer and nanoparticle production: Current status and challenges. Can. J. Chem. Eng..

[65] Ge Y., Li Z. (2018). Application of lignin and its derivatives in adsorption of heavy metal ions in water: A review. ACS Sustain. Chem. Eng..

[66] Agustiany E.A., Rasyidur Ridho M., Rahmi DN M., Madyaratri E.W., Falah F., Lubis M.A.R., Solihat N.N., Syamani F.A., Karungamye P., Sohail A. (2022). Recent developments in lignin modification and its application in lignin-based green composites: A review. Polym. Compos..

[67] Dahiya S., Katakojwala R., Ramakrishna S., Mohan S.V. (2020). Biobased products and life cycle assessment in the context of circular economy and sustainability. Mater. Circ. Econ..

[68] Pham C.D., Dang M.D., Ly T.B., Tran K.D., Vo N.T., Do N.H., Mai P.T., Le P.K. (2023). A review of the extraction methods and advanced applications of lignin-silica hybrids derived from natural sources. Int. J. Biol. Macromol..

[69] Duarah P., Haldar D., Purkait M.K. (2020). Technological advancement in the synthesis and applications of lignin-based nanoparticles derived from agro-industrial waste residues: A review. Int. J. Biol. Macromol..

[70] Crini G. (2005). Recent developments in polysaccharide-based materials used as adsorbents in wastewater treatment. Prog. Polym. Sci..

[71] Guerra F.D., Attia M.F., Whitehead D.C., Alexis F. (2018). Nanotechnology for environmental remediation: Materials and applications. Molecules.

[72] Ayadi A., Jellouli Ennigrou D., Proietto F., Hamzaoui A.H., Jaouadi M. (2023). Electrochemical Degradation of Phenol in Aqueous Solutions Using Activated Carbon-ZnO Composite. Environ. Eng. Sci..

[73] Zhao Y., Wang X., Li D., Tang H., Yu D., Wang L., Jiang L. (2020). Effect of anionic polysaccharides on conformational changes and antioxidant properties of protein-polyphenol binary covalently-linked complexes. Process Biochem..

[74] De Melo L.F.M., Martins V.G.d.Q.A., da Silva A.P., de Oliveira Rocha H.A., Scortecci K.C. (2023). Biological and pharmacological aspects of tannins and potential biotechnological applications. Food Chem..

[75] Nobahar A., Carlier J.D., Miguel M.G., Costa M.C. (2021). A review of plant metabolites with metal interaction capacity: A green approach for industrial applications. BioMetals.

[76] Awad A.M., Shaikh S.M., Jalab R., Gulied M.H., Nasser M.S., Benamor A., Adham S. (2019). Adsorption of organic pollutants by natural and modified clays: A comprehensive review. Sep. Purif. Technol..

[77] Zheng X., Zhang J., Wang J., Qi X., Rosenholm J.M., Cai K. (2015). Polydopamine coatings in confined nanopore space: Toward improved retention and release of hydrophilic cargo. J. Phys. Chem. C.

[78] Rzepecka-Stojko A., Stojko J., Kurek-Górecka A., Górecki M., Kabała-Dzik A., Kubina R., Moździerz A., Buszman E. (2015). Polyphenols from bee pollen: Structure, absorption, metabolism and biological activity. Molecules.

[79] Wu L., Du C., He J., Yang Z., Li H. (2020). Effective adsorption of diclofenac sodium from neutral aqueous solution by low-cost lignite activated cokes. J. Hazard. Mater..

[80] Ma C.-Y., Xu L.-H., Sun Q., Shen X.-J., Wen J.-L., Yuan T.-Q. (2022). Tailored one-pot lignocellulose fractionation to maximize biorefinery toward controllable producing lignin nanoparticles and facilitating enzymatic hydrolysis. Chem. Eng. J..

[81] Zhang W., Qiu X., Wang C., Zhong L., Fu F., Zhu J., Zhang Z., Qin Y., Yang D., Xu C.C. (2022). Lignin derived carbon materials: Current status and future trends. Carbon Res..

[82] Grabber J.H. (2005). How do lignin composition, structure, and cross-linking affect degradability? A review of cell wall model studies. Crop Sci..

[83] Khalil H.A., Bhat A., Yusra A.I. (2012). Green composites from sustainable cellulose nanofibrils: A review. Carbohydr. Polym..

[84] Ma Q., Cui L., Zhou S., Li Y., Shi W., Ai S. (2018). Iron nanoparticles in situ encapsulated in lignin-derived hydrochar as an effective catalyst for phenol removal. Environ. Sci. Pollut. Res..

[85] Custodis V.B., Karakoulia S.A., Triantafyllidis K.S., van Bokhoven J.A. (2016). Catalytic fast pyrolysis of lignin over high-surface-area mesoporous aluminosilicates: Effect of porosity and acidity. ChemSusChem.

[86] HPS A.K., Saurabh C.K., Adnan A., Fazita M.N., Syakir M., Davoudpour Y., Rafatullah M., Abdullah C., Haafiz M., Dungani R. (2016). A review on chitosan-cellulose blends and nanocellulose reinforced chitosan biocomposites: Properties and their applications. Carbohydr. Polym..

[87] Liu Z.-H., Hao N., Shinde S., Pu Y., Kang X., Ragauskas A.J., Yuan J.S. (2019). Defining lignin nanoparticle properties through tailored lignin reactivity by sequential organosolv fragmentation approach (SOFA). Green Chem..

[88] Shangguan J., Hensley A.J., Gradiski M.V., Pfriem N., McEwen J.-S., Morris R.H., Chin Y.-H.C. (2020). The role of protons and hydrides in the catalytic hydrogenolysis of guaiacol at the ruthenium nanoparticle–water interface. ACS Catal..

[89] Ersöz A., Denizli A., Şener İ., Atılır A., Diltemiz S., Say R. (2004). Removal of phenolic compounds with nitrophenol-imprinted polymer based on π–π and hydrogen-bonding interactions. Sep. Purif. Technol..

[90] Mohanapriya V., Sakthivel R., Pham N.D.K., Cheng C.K., Le H.S., Dong T.M.H. (2023). Nanotechnology-A ray of hope for heavy metals removal. Chemosphere.

[91] Dutta S., Bhaumik A., Wu K.C.-W. (2014). Hierarchically porous carbon derived from polymers and biomass: Effect of interconnected pores on energy applications. Energy Environ. Sci..

[92] Pandey A., Kalamdhad A., Sharma Y.C. (2023). Recent advances of nanocellulose as biobased adsorbent for heavy metal ions removal: A sustainable approach integrating with waste management. Environ. Nanotechnol. Monit. Manag..

[93] Sohni S., Hassan T., Khan S.B., Akhtar K., Bakhsh E.M., Hashim R., Nidaullah H., Khan M., Khan S.A. (2023). Lignin nanoparticles-reduced graphene oxide based hydrogel: A novel strategy for environmental applications. Int. J. Biol. Macromol..

[94] Selvasembian R., Singh P. (2022). Biosorption for Wastewater Contaminants.

[95] Du B., Chai L., Wang Y., Wang X., Chen X., Zhou J., Sun R.-C. (2023). Fabrication of demethylated lignin-based micro-particle for efficient adsorption of malachite green from aqueous solution. J. Mol. Liq..

[96] Sohni S., Hashim R., Nidaullah H., Lamaming J., Sulaiman O. (2019). Chitosan/nano-lignin based composite as a new sorbent for enhanced removal of dye pollution from aqueous solutions. Int. J. Biol. Macromol..

[97] Ahmad K., Ghatak H.R., Ahuja S. (2020). A review on photocatalytic remediation of environmental pollutants and H2 production through water splitting: A sustainable approach. Environ. Technol. Innov..

[98] Sinha V., Chakma S. (2019). Advances in the preparation of hydrogel for wastewater treatment: A concise review. J. Environ. Chem. Eng..

[99] Li Q., Zhan Z., Jin S., Tan B. (2017). Wettable magnetic hypercrosslinked microporous nanoparticle as an efficient adsorbent for water treatment. Chem. Eng. J..

[100] Meng Y., Liu T., Yu S., Cheng Y., Lu J., Wang H. (2020). A lignin-based carbon aerogel enhanced by graphene oxide and application in oil/water separation. Fuel.

[101] Liu J., Jiang J., Meng Y., Aihemaiti A., Xu Y., Xiang H., Gao Y., Chen X. (2020). Preparation, environmental application and prospect of biochar-supported metal nanoparticles: A review. J. Hazard. Mater..

[102] Muthukumaran P., Babu P.S., Shyamalagowri S., Aravind J., Kamaraj M., Govarthanan M. (2022). Polymeric biomolecules based nanomaterials: Production strategies and pollutant mitigation as an emerging tool for environmental application. Chemosphere.

[103] Dey N., Vickram S., Thanigaivel S., Subbaiya R., Kim W., Karmegam N., Govarthanan M. (2022). Nanomaterials for transforming barrier properties of lignocellulosic biomass towards potential applications–A review. Fuel.

[104] Rahman Z., Singh V.P. (2019). The relative impact of toxic heavy metals (THMs)(arsenic (As), cadmium (Cd), chromium (Cr)(VI), mercury (Hg), and lead (Pb)) on the total environment: An overview. Environ. Monit. Assess..

[105] Senila M. (2023). Metal and metalloid monitoring in water by passive sampling–A review. Rev. Anal. Chem..

[106] Senila M. (2024). Recent Advances in the Determination of Major and Trace Elements in Plants Using Inductively Coupled Plasma Optical Emission Spectrometry. Molecules.

[107] Vardhan K.H., Kumar P.S., Panda R.C. (2019). A review on heavy metal pollution, toxicity and remedial measures: Current trends and future perspectives. J. Mol. Liq..

[108] Sharma R., Agrawal P.R., Kumar R., Ittishree, Gupta G. (2022). Biosorption for Eliminating Inorganic Contaminants (IOCs) from Wastewater. Biosorption for Wastewater Contaminants.

[109] Yang X., Wan Y., Zheng Y., He F., Yu Z., Huang J., Wang H., Ok Y.S., Jiang Y., Gao B. (2019). Surface functional groups of carbon-based adsorbents and their roles in the removal of heavy metals from aqueous solutions: A critical review. Chem. Eng. J..

[110] Kayan G.Ö., Kayan A. (2021). Composite of natural polymers and their adsorbent properties on the dyes and heavy metal ions. J. Polym. Environ..

[111] Yoosaf K., Ipe B.I., Suresh C.H., Thomas K.G. (2007). In situ synthesis of metal nanoparticles and selective naked-eye detection of lead ions from aqueous media. J. Phys. Chem. C.

[112] Feng M., Zhang P., Zhou H.-C., Sharma V.K. (2018). Water-stable metal-organic frameworks for aqueous removal of heavy metals and radionuclides: A review. Chemosphere.

[113] Amonette J.E., Joseph S. (2012). Characteristics of biochar: Microchemical properties. Biochar for Environmental Management.

[114] Luo M., Lin H., Li B., Dong Y., He Y., Wang L. (2018). A novel modification of lignin on corncob-based biochar to enhance removal of cadmium from water. Bioresour. Technol..

[115] Santander P., Butter B., Oyarce E., Yáñez M., Xiao L.-P., Sánchez J. (2021). Lignin-based adsorbent materials for metal ion removal from wastewater: A review. Ind. Crops Prod..

[116] Mukhopadhyay A., Duttagupta S., Mukherjee A. (2022). Emerging organic contaminants in global community drinking water sources and supply: A review of occurrence, processes and remediation. J. Environ. Chem. Eng..

[117] Arun J., Nirmala N., Priyadharsini P., Dawn S., Santhosh A., Gopinath K., Govarthanan M. (2022). A mini-review on bioderived carbon and its nanocomposites for removal of organic pollutants from wastewater. Mater. Lett..

[118] Zhang B., Fang C., Ning J., Dai R., Liu Y., Wu Q., Zhang F., Zhang W., Dou S., Liu X. (2023). Unusual aliovalent Cd doped γ-Bi2MoO6 nanomaterial for efficient photocatalytic degradation of sulfamethoxazole and rhodamine B under visible light irradiation. Carbon Neutralization.

[119] Zhang B., Liu G., Shi H., Wu Q., Xue S., Shao T., Zhang F., Liu X. (2023). Density functional theory study of electronic structure and optical properties of ln3+-doped γ-bi2moo6 (ln= gd, ho, yb). Crystals.

[120] Kumar R., Sudhaik A., Raizada P., Hosseini-Bandegharaei A., Thakur V.K., Saini A., Saini V., Singh P. (2020). An overview on bismuth molybdate based photocatalytic systems: Controlled morphology and enhancement strategies for photocatalytic water purification. J. Environ. Chem. Eng..

[121] Li K., Liu C., Li J., Wang G., Wang K. (2024). Architecting inorganic/organic S-scheme heterojunction of Bi_4_Ti_3_O1_2_ coupling with g-C3N4 for photocatalytic H2O2 production from pure water. Acta Phys.-Chim. Sin..

[122] Shao X., Li K., Li J., Cheng Q., Wang G., Wang K. (2023). Investigating S-scheme charge transfer pathways in NiS@Ta_2_O_5_ hybrid nanofibers for photocatalytic CO2 conversion. Chin. J. Catal..

[123] Pasini S.M., Valerio A., Yin G., Wang J., de Souza S.M.G.U., Hotza D., de Souza A.A.U. (2021). An overview on nanostructured TiO_2_–containing fibers for photocatalytic degradation of organic pollutants in wastewater treatment. J. Water Process Eng..

[124] Tan X.-F., Zhu S.-S., Wang R.-P., Chen Y.-D., Show P.-L., Zhang F.-F., Ho S.-H. (2021). Role of biochar surface characteristics in the adsorption of aromatic compounds: Pore structure and functional groups. Chin. Chem. Lett..

[125] Pandey S., Mandari K.K., Kim J., Kang M., Fosso-Kankeu E. (2020). Recent advancement in visible-light-responsive photocatalysts in heterogeneous photocatalytic water treatment technology. Photocatalysts in Advanced Oxidation Processes for Wastewater Treatment.

[126] Schneider W.D.H., Dillon A.J.P., Camassola M. (2021). Lignin nanoparticles enter the scene: A promising versatile green tool for multiple applications. Biotechnol. Adv..

[127] Saravanan A., Swaminaathan P., Kumar P.S., Yaashikaa P., Kamalesh R., Rangasamy G. (2023). A comprehensive review on immobilized microbes-biochar and their environmental remediation: Mechanism, challenges and future perspectives. Environ. Res..

[128] Uddin M.M., Zakeel M.C.M., Zavahir J.S., Marikar F.M., Jahan I. (2021). Heavy metal accumulation in rice and aquatic plants used as human food: A general review. Toxics.

[129] Di Giulio M., Holderegger R., Tobias S. (2009). Effects of habitat and landscape fragmentation on humans and biodiversity in densely populated landscapes. J. Environ. Manag..

[130] Sharma A., Anjana, Rana H., Goswami S. (2022). A comprehensive review on the heavy metal removal for water remediation by the application of lignocellulosic biomass-derived nanocellulose. J. Polym. Environ..

[131] Wu Y., Guan C.-Y., Griswold N., Hou L.-y., Fang X., Hu A., Hu Z.-q., Yu C.-P. (2020). Zero-valent iron-based technologies for removal of heavy metal (loid) s and organic pollutants from the aquatic environment: Recent advances and perspectives. J. Clean. Prod..

[132] Iravani S., Varma R.S. (2020). Greener synthesis of lignin nanoparticles and their applications. Green Chem..

[133] Wang L., Rinklebe J., Tack F.M., Hou D. (2021). A review of green remediation strategies for heavy metal contaminated soil. Soil Use Manag..

[134] Bolan N., Hoang S.A., Beiyuan J., Gupta S., Hou D., Karakoti A., Joseph S., Jung S., Kim K.-H., Kirkham M. (2022). Multifunctional applications of biochar beyond carbon storage. Int. Mater. Rev..

[135] Kacprzak M., Kupich I., Jasinska A., Fijalkowski K. (2022). Bio-based waste’substrates for degraded soil improvement—Advantages and challenges in European context. Energies.

[136] Zhu J., Agarwal U.P., Ciesielski P.N., Himmel M.E., Gao R., Deng Y., Morits M., Österberg M. (2021). Towards sustainable production and utilization of plant-biomass-based nanomaterials: A review and analysis of recent developments. Biotechnol. Biofuels.

[137] Evans S., Vladimirova D., Holgado M., van Fossen K., Yang M., Silva E.A., Barlow C.Y. (2017). Business model innovation for sustainability: Towards a unified perspective for creation of sustainable business models. Bus. Strategy Environ..

[138] Ganie A.S., Bano S., Khan N., Sultana S., Rehman Z., Rahman M.M., Sabir S., Coulon F., Khan M.Z. (2021). Nanoremediation technologies for sustainable remediation of contaminated environments: Recent advances and challenges. Chemosphere.

[139] Qian Y., Qin C., Chen M., Lin S. (2020). Nanotechnology in soil remediation− applications vs. implications. Ecotoxicol. Environ. Saf..

[140] Demirer G.S., Silva T.N., Jackson C.T., Thomas J.B., Ehrhardt D.W., Rhee S.Y., Mortimer J.C., Landry M.P. (2021). Nanotechnology to advance CRISPR–Cas genetic engineering of plants. Nat. Nanotechnol..

[141] Yadav A., Yadav K., Ahmad R., Abd-Elsalam K.A. (2023). Emerging Frontiers in Nanotechnology for Precision Agriculture: Advancements, Hurdles and Prospects. Agrochemicals.

[142] Xu D.-M., Fu R.-B., Wang J.-X., Shi Y.-X., Guo X.-P. (2021). Chemical stabilization remediation for heavy metals in contaminated soils on the latest decade: Available stabilizing materials and associated evaluation methods-A critical review. J. Clean. Prod..

[143] Zhu H., Luo W., Ciesielski P.N., Fang Z., Zhu J., Henriksson G., Himmel M.E., Hu L. (2016). Wood-derived materials for green electronics, biological devices, and energy applications. Chem. Rev..

[144] Nguyen V.-H., Smith S.M., Wantala K., Kajitvichyanukul P. (2020). Photocatalytic remediation of persistent organic pollutants (POPs): A review. Arab. J. Chem..

[145] Aven T., Zio E. (2014). Foundational issues in risk assessment and risk management. Risk Anal..

[146] Santo Pereira A.d.E., de Oliveira J.L., Savassa S.M., Rogério C.B., de Medeiros G.A., Fraceto L.F. (2022). Lignin nanoparticles: New insights for a sustainable agriculture. J. Clean. Prod..

[147] Zhang Z., Cissoko N., Wo J., Xu X. (2009). Factors influencing the dechlorination of 2,4-dichlorophenol by Ni–Fe nanoparticles in the presence of humic acid. J. Hazard. Mater..

[148] HogenEsch H., O’Hagan D.T., Fox C.B. (2018). Optimizing the utilization of aluminum adjuvants in vaccines: You might just get what you want. npj Vaccines.

[149] Haynes H., Asmatulu R. (2013). Nanotechnology safety in the aerospace industry. Nanotechnology Safety.

[150] Saleem H., Zaidi S.J. (2020). Nanoparticles in reverse osmosis membranes for desalination: A state of the art review. Desalination.

[151] Jaldurgam F.F., Ahmad Z., Touati F. (2021). Synthesis and performance of large-scale cost-effective environment-friendly nanostructured thermoelectric materials. Nanomaterials.

[152] Candan Z., Tozluoglu A., Gonultas O., Yildirim M., Fidan H., Alma M.H., Salan T. (2022). Nanocellulose: Sustainable biomaterial for developing novel adhesives and composites. Industrial Applications of Nanocellulose and Its Nanocomposites.

[153] Khaddage F., Müller W., Flintoff K. (2016). Advancing mobile learning in formal and informal settings via mobile app technology: Where to from here, and how?. J. Educ. Technol. Soc..

[154] Stavis S.M., Fagan J.A., Stopa M., Liddle J.A. (2018). Nanoparticle manufacturing–heterogeneity through processes to products. ACS Appl. Nano Mater..

[155] Cortese A.D. (2003). The critical role of higher education in creating a sustainable future. Plan. High. Educ..

[156] Kinzig A.P. (2001). Bridging disciplinary divides to address environmental and intellectual challenges. Ecosystems.

[157] Livingston A., Trout B.L., Horvath I.T., Johnson M.D., Vaccaro L., Coronas J., Babbitt C.W., Zhang X., Pradeep T., Drioli E. (2020). Challenges and directions for green chemical engineering—Role of nanoscale materials. Sustainable Nanoscale Engineering.

[158] Acciardo E., Tabasso S., Cravotto G., Bensaid S. (2022). Process intensification strategies for lignin valorization. Chem. Eng. Process.Process Intensif..

[159] Braithwaite J., Churruca K., Long J.C., Ellis L.A., Herkes J. (2018). When complexity science meets implementation science: A theoretical and empirical analysis of systems change. BMC Med..

[160] Viswanathan M., Sridharan S. (2012). Product Development for the BoP: Insights on Concept and Prototype Development from University-Based Student Projects in I ndia. J. Prod. Innov. Manag..

[161] Gorelick S.M., Zheng C. (2015). Global change and the groundwater management challenge. Water Resour. Res..

[162] Dragone G., Kerssemakers A.A., Driessen J.L., Yamakawa C.K., Brumano L.P., Mussatto S.I. (2020). Innovation and strategic orientations for the development of advanced biorefineries. Bioresour. Technol..

[163] Simenstad C., Tanner C., Crandell C., White J., Cordell J. (2005). Challenges of habitat restoration in a heavily urbanized estuary: Evaluating the investment. J. Coast. Res..

[164] Zamora-Ledezma C., Negrete-Bolagay D., Figueroa F., Zamora-Ledezma E., Ni M., Alexis F., Guerrero V.H. (2021). Heavy metal water pollution: A fresh look about hazards, novel and conventional remediation methods. Environ. Technol. Innov..

[165] Sharma P., Bano A., Singh S.P., Sharma S., Xia C., Nadda A.K., Lam S.S., Tong Y.W. (2022). Engineered microbes as effective tools for the remediation of polyaromatic aromatic hydrocarbons and heavy metals. Chemosphere.

[166] Kai D., Tan M.J., Chee P.L., Chua Y.K., Yap Y.L., Loh X.J. (2016). Towards lignin-based functional materials in a sustainable world. Green Chem..

[167] Kylili A., Koutinas M., Georgali P.-Z., Fokaides P.A. (2023). Lignin valorisation: Life Cycle Assessment (LCA) considerations for enabling Circular Bioeconomy. Int. J. Sustain. Energy.

[168] Kumar B., Verma P. (2021). Biomass-based biorefineries: An important architype towards a circular economy. Fuel.

[169] Morganti P., Danti S., Coltelli M.B. (2018). Chitin and lignin to produce biocompatible tissues. Res. Clin. Dermatol..

[170] Wertz J.-L., Mengal P., Perez S. (2022). Biomass in the Bioeconomy: Focus on the EU and US.

[171] Fabbri F., Bischof S., Mayr S., Gritsch S., Jimenez Bartolome M., Schwaiger N., Guebitz G.M., Weiss R. (2023). The Biomodified Lignin Platform: A Review. Polymers.

[172] Manjunatha S., Biradar D., Aladakatti Y.R. (2016). Nanotechnology and its applications in agriculture: A review. J. Farm. Sci..

